# Supplementary material for: The chromatin landscape of pathogenic transcriptional cell states in rheumatoid arthritis
Source: Nat Commun. 2024 May 31;15:4650. doi: 10.1038/s41467-024-48620-7 (PMC11143375; doi:10.1038/s41467-024-48620-7)
Supplement: Supplementary file 1 — Editorial Summary [file 41467_2024_48620_MOESM1_ESM.docx]

**Editorial Summary**

The epigenetic changes underlying the heterogeneity of RA disease presentation have been the subject of intense scrutiny. In this study, the authors use multiple single-cell sequencing datasets to define ‘chromatin superstates’ in patients with RA, which associate with distinct transcription factors and disease phenotypes.

**Peer review information**: *Nature Communications* thanks Caroline Ospelt, Richard Scheuermann and the other, anonymous, reviewer(s) for their contribution to the peer review of this work. A peer review file is available.
