# Supplementary material for: The chromatin landscape of pathogenic transcriptional cell states in rheumatoid arthritis
Source: Nat Commun. 2024 May 31;15:4650. doi: 10.1038/s41467-024-48620-7 (PMC11143375; doi:10.1038/s41467-024-48620-7)
Supplement: Supplementary file 2 — Supplementary Information [file 41467_2024_48620_MOESM2_ESM.pdf]

The chromatin landscape of pathogenic transcriptional cell states in rheumatoid arthritis

Kathryn Weinand, Saori Sakaue, Aparna Nathan, Anna Helena Jonsson, Fan Zhang, Gerald F. M. Watts, Majd Al Suqri, Zhu Zhu, Accelerating Medicines Partnership Program: Rheumatoid Arthritis and Systemic Lupus Erythematosus (AMP RA/SLE) Network, Deepak A. Rao, Jennifer H. Anolik, Michael B. Brenner, Laura T. Donlin, Kevin Wei, Soumya Raychaudhuri

Supplementary Figures and Tables

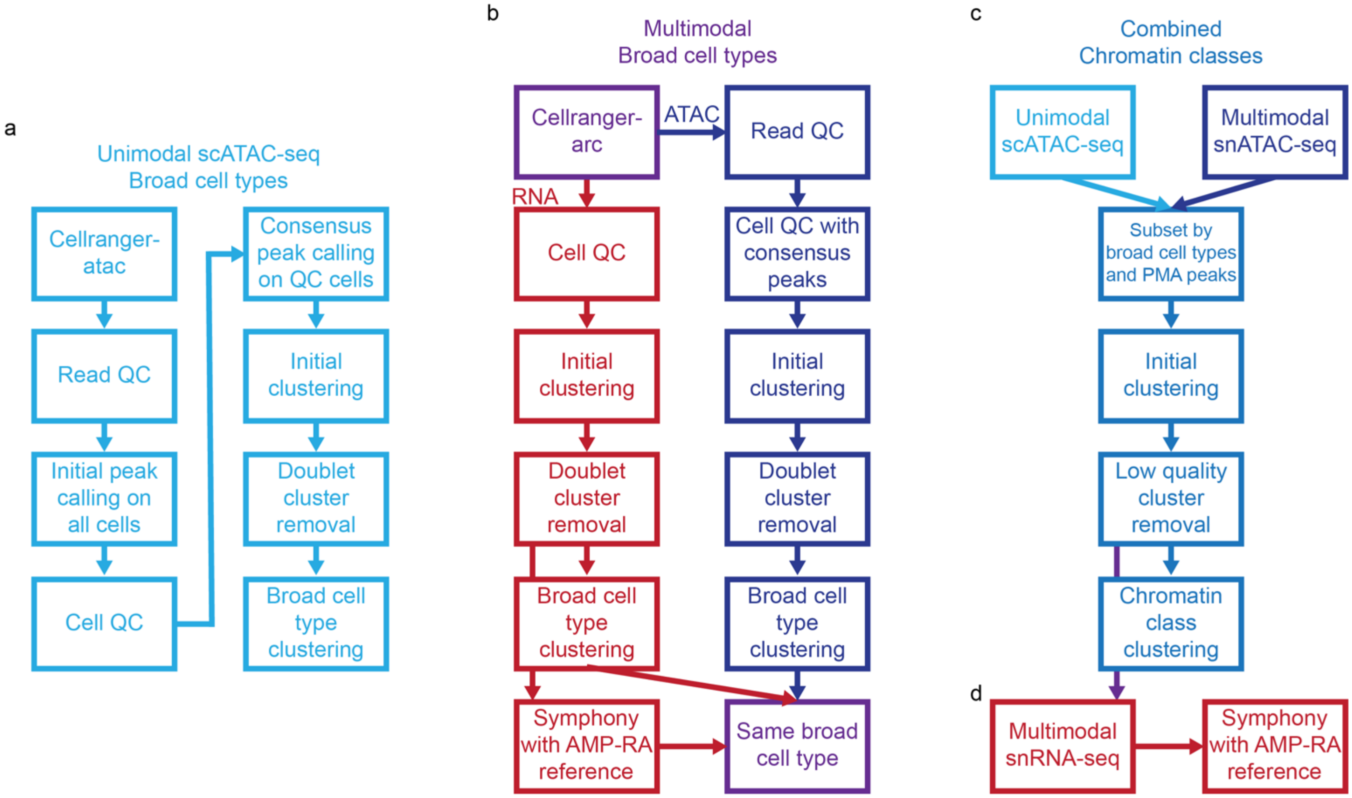

Supplementary Fig. 1. Computational methods workflows for cell type, chromatin class, and transcriptional cell state annotations.

a. Unimodal scATAC-seq (light blue) workflow from 10x Genomics Cell Ranger ATAC 1.1.0 to broad cell type clustering.

b. Multiome (purple) workflow for both snATAC-seq (dark blue) and snRNA-seq (red) from 10x Genomics Cell Ranger ARC 2.0.0 to broad cell type clustering. In addition to calling broad cell types within each modality, we used the non-doublet multimodal snRNA-seq cells as a query dataset to map onto the AMP-RA synovial tissue CITE-seq broad cell type reference[14] using Symphony[95]. We removed any cells whose broad cell types did not match for all three annotations (snATAC-seq broad cell type, snRNA-seq broad cell type, and classified AMP-RA broad cell type).

c. Chromatin class (middle blue) workflow from combining unimodal scATAC-seq and multimodal snATAC-seq at the broad cell type level to chromatin class clustering.

d. Transcriptional cell state workflow using multimodal snRNA-seq cells as queries to map onto the AMP-RA synovial tissue CITE-seq references[14] using Symphony[95]. This is done for each cell type using the cells with chromatin class annotations.

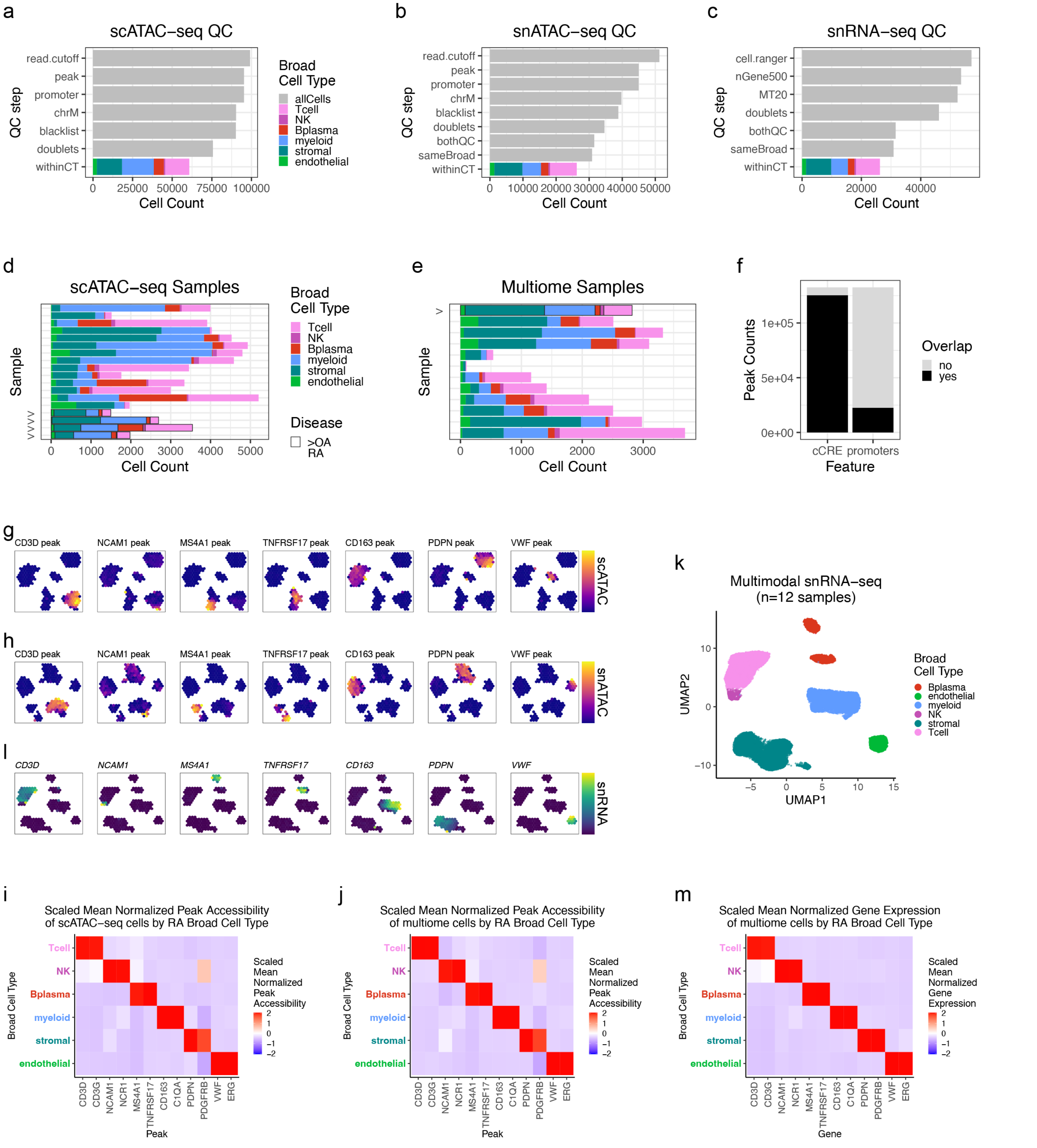

Supplementary Fig. 2. Quality Control Measures and Broad Cell Type Markers.
a.-c. Quality control steps ending in the final cell counts for all broad cell types in (**a.**) unimodal scATAC-seq, (**b.**) multimodal snATAC-seq, and (**c.**) multimodal snRNA-seq datasets.
d.-e. Broad cell type cell counts per sample for (**d.**) unimodal and (**e.**) multimodal datasets. OA samples are highlighted with a '>' symbol and black bar outlines.
f. Number of trimmed open chromatin peaks overlapping ENCODE cCREs[25] and promoters[26].

g.-h. Binned normalized marker peak accessibility visualized across broad cell types on the corresponding UMAPs for (**g.**) unimodal scATAC-seq and (**h.**) multimodal snATAC-seq datasets.
i.-j. Scaled mean normalized marker peak accessibility across broad cell types for (**i.**) unimodal scATAC-seq and (**j.**) multimodal snATAC-seq datasets.
k. Broad cell type identification for multiome snRNA-seq cells visualized on a UMAP.
l. Binned normalized marker gene expression visualized across broad cell types on the corresponding UMAP for multimodal snRNA-seq datasets.
m. Scaled mean normalized marker gene expression across broad cell types for multimodal snRNA-seq datasets.

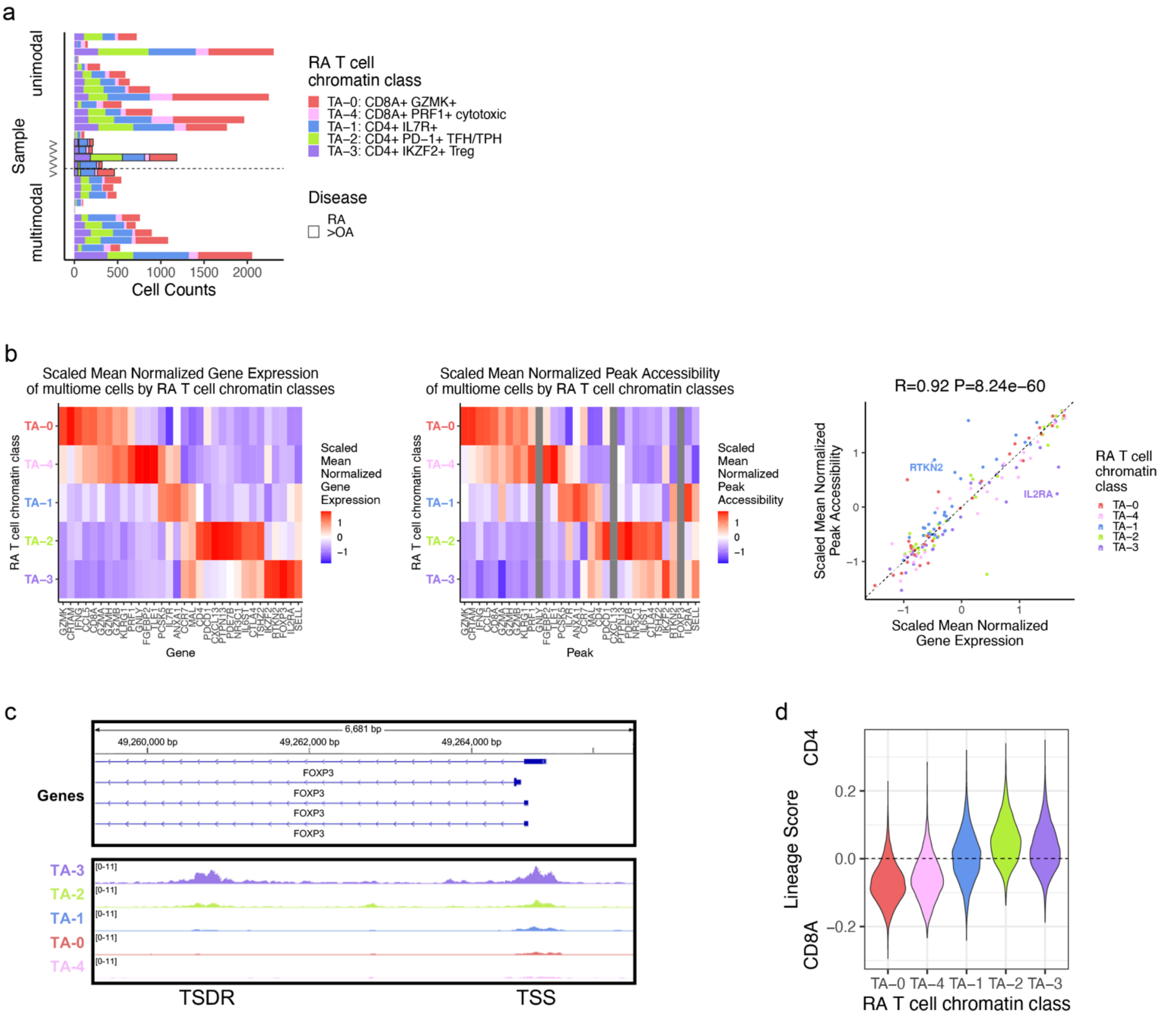

Supplementary Fig. 3. Further analyses of RA T cell chromatin classes.

a. T cell chromatin class cell counts per sample for unimodal and multimodal datasets. OA samples are highlighted with a '>' symbol and black bar outlines.

b. Scaled mean normalized marker gene expression (**left**) and peak accessibility (**middle**) across T cell chromatin classes in multimodal datasets and the Pearson correlation (R) and two-sided p-value (P) between them (**right**). Genes without an overlapping promoter peak are colored in grey in the **middle** panel. On the **right**, correlation calculated from 145 gene/class combinations.

c. FOXP3 locus (chrX:49,259,337-49,266,016) with selected gene isoforms and chromatin accessibility reads aggregated by chromatin class and scaled by read counts per class (**Methods**). Transcriptional start site (TSS) and Treg-specific demethylated region[29] (TSDR) highlighted as areas of selective open chromatin in T_A-3: CD4+ IKZF2+ Tregs.

d. Lineage score by cell based on normalized peak accessibility for T cell lineage-associated promoter peaks (**Methods**), segregated by T cell chromatin classes.

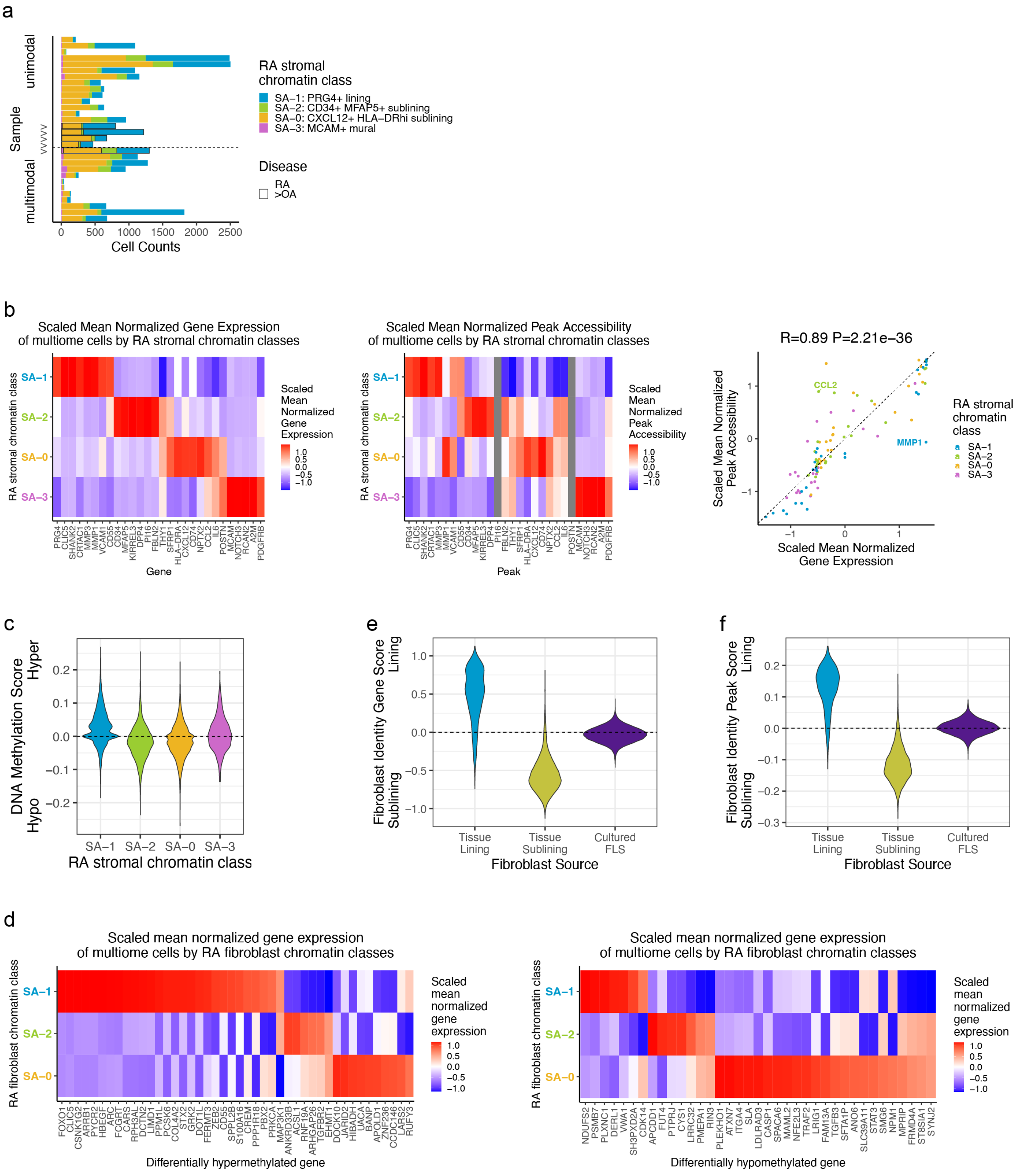

Supplementary Fig. 4. Further analyses of RA stromal chromatin classes.
a. Stromal chromatin class cell counts per sample for unimodal and multimodal datasets. OA samples are highlighted with a '>' symbol and black bar outlines.
b. Scaled mean normalized marker gene expression (**left**) and peak accessibility (**middle**) across stromal chromatin classes in multimodal datasets and the Pearson correlation (R) and two-sided p-value (P) between them (**right**). Genes without an overlapping promoter peak are colored in grey in the

middle panel. On the **right**, correlation calculated from 104 gene/class combinations.
c. DNA methylation score by cell based on normalized peak accessibility for differential DNA methylation-associated promoter peaks (**Methods**), segregated by stromal chromatin classes. Differential methylation was calculated for RA FLS cell lines compared to OA FLS cell lines and obtained from Nakano et al., Ann Rheum Dis, 2013[47].
d. Scaled mean normalized gene expression across fibroblast chromatin classes in multimodal datasets for genes associated with hypermethylated (**left**) and hypomethylated (**right**) differentially methylated regions (DMR).
e-f. Fibroblast identity score between lining and sublining tissue fibroblasts by cell based on (**e.**) normalized gene expression for differentially expressed genes and (**f.**) normalized peak accessibility for differentially accessible peaks (**Methods**), segregated by fibroblast source. The cultured fibroblast-like synoviocyte (FLS) data was obtained from Smith et al., Nat Immunol, 2023[44]. All pairwise combinations of scores by source were significantly different by two-sided Wilcoxon test within both gene- and peak-based metrics.

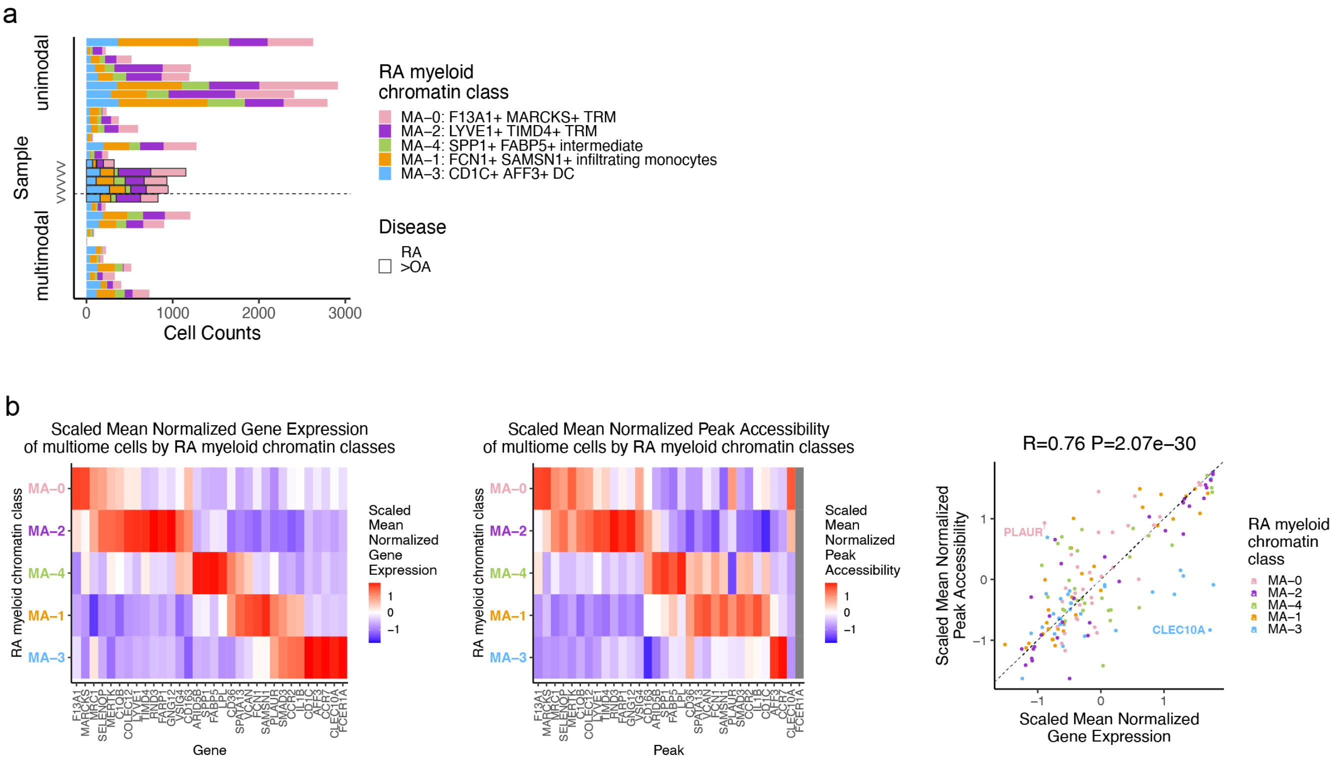

Supplementary Fig. 5. Further analyses of RA myeloid chromatin classes.
a. Myeloid chromatin class cell counts per sample for unimodal and multimodal datasets. OA samples are highlighted with a '>' symbol and black bar outlines.
b. Scaled mean normalized marker gene expression (**left**) and peak accessibility (**middle**) across myeloid chromatin classes in multimodal datasets and the Pearson correlation (R) and two-sided p-value (P) between them (**right**). Genes without an overlapping promoter peak are colored in grey in the **middle** panel. On the **right**, correlation calculated from 155 gene/class combinations.

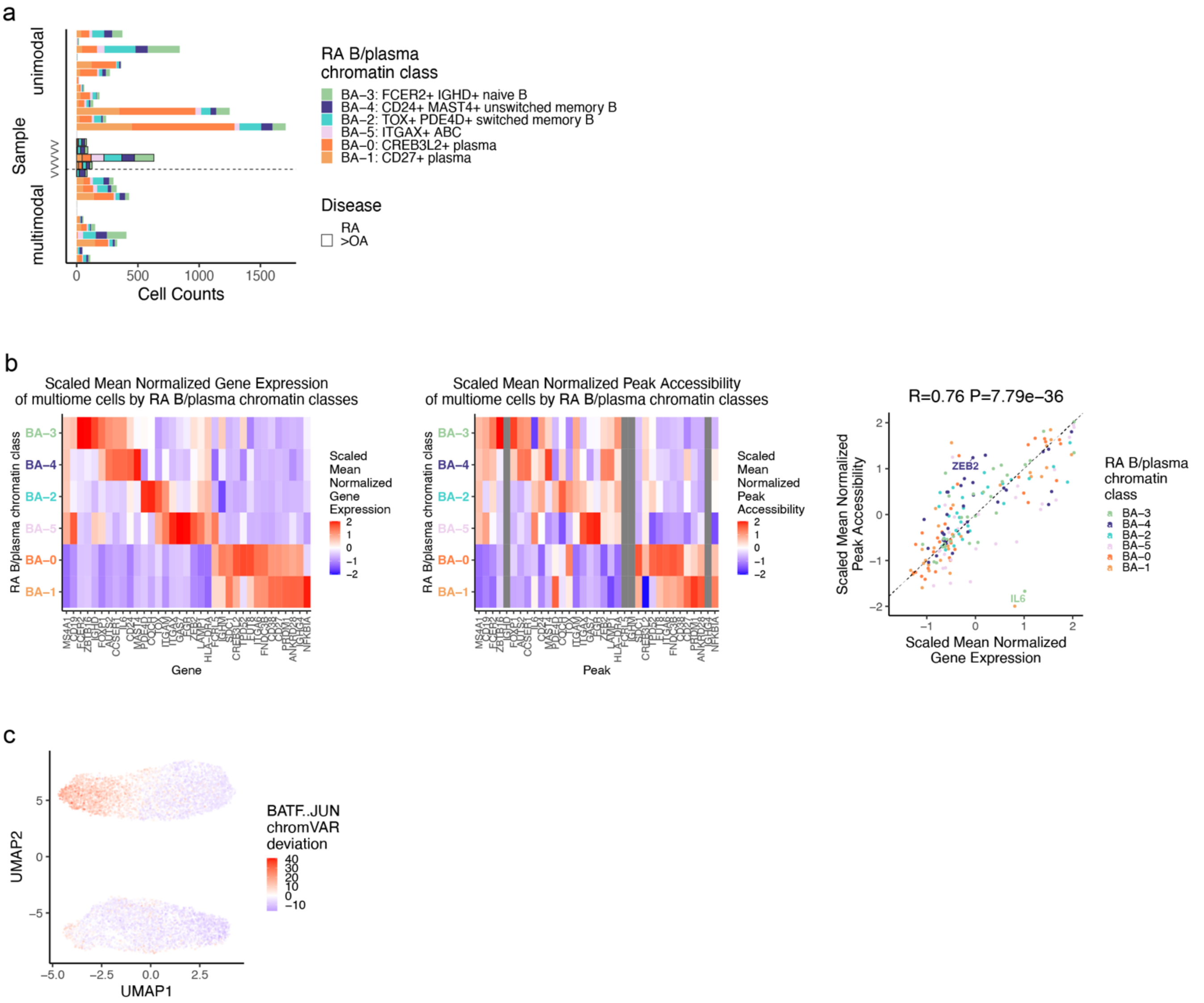

Supplementary Fig. 6. Further analyses of RA B/plasma chromatin classes.
a. B/plasma chromatin class cell counts per sample for unimodal and multimodal datasets. OA samples are highlighted with a '>' symbol and black bar outlines.
b. Scaled mean normalized marker gene expression (**left**) and peak accessibility (**middle**) across B/plasma chromatin classes in multimodal datasets and the Pearson correlation (R) and two-sided p-value (P) between them (**right**). Genes without an overlapping promoter peak are colored in grey in the **middle** panel. On the **right**, correlation calculated from 186 gene/class combinations.
c. UMAP colored by chromVAR[34] deviations for the BATF..JUN motif.

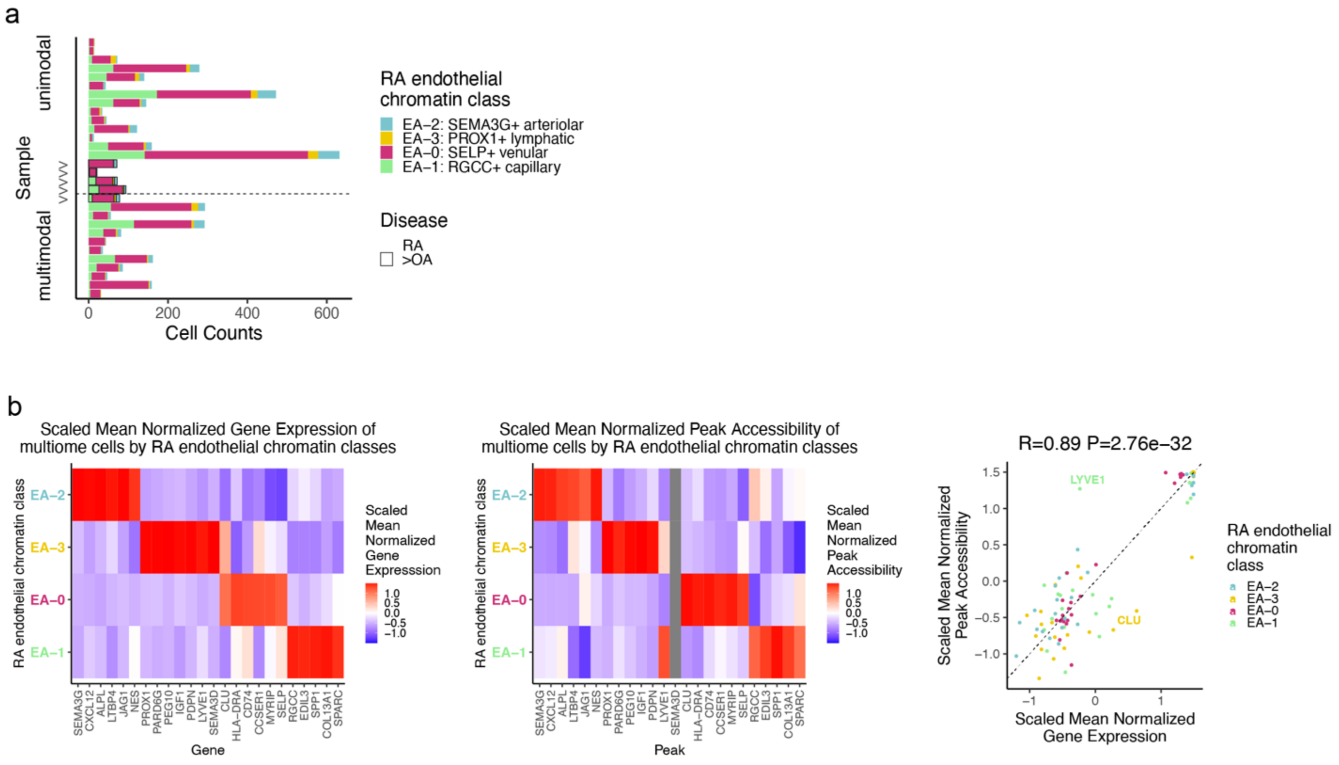

Supplementary Fig. 7. Further analyses of RA endothelial chromatin classes.
a. Endothelial chromatin class cell counts per sample for unimodal and multimodal datasets. OA samples are highlighted with a '>' symbol and black bar outlines.
b. Scaled mean normalized marker gene expression (**left**) and peak accessibility (**middle**) across endothelial chromatin classes in multimodal datasets and the Pearson correlation (R) and two-sided p-value (P) between them (**right**). Genes without an overlapping promoter peak are colored in grey in the **middle** panel. On the **right**, correlation calculated from 92 gene/class combinations.

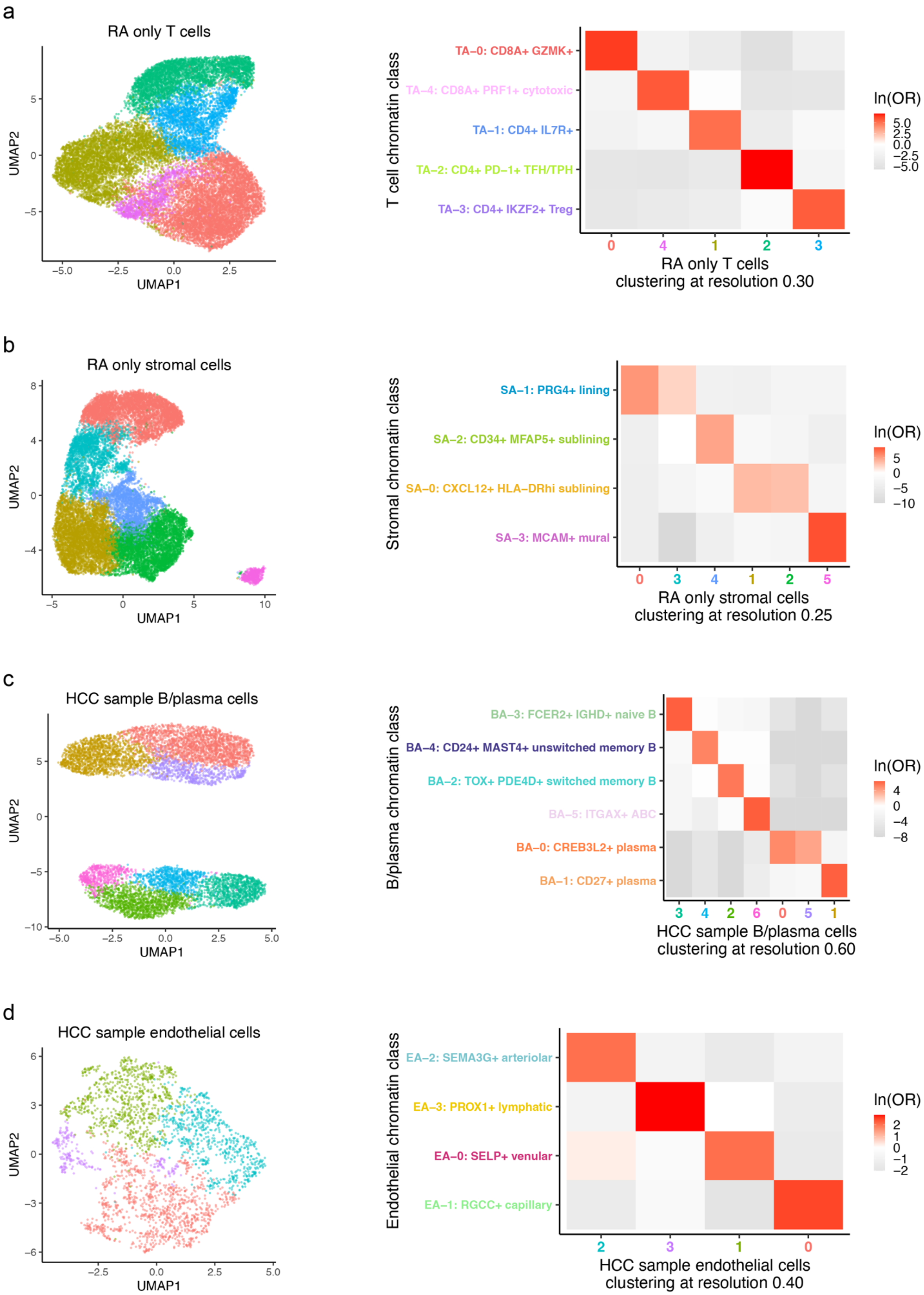

a
RA only T cells
UMAP2
UMAP1
T cell chromatin class
TA–0: CD8A+ GZMK+
TA–4: CD8A+ PRF1+ cytotoxic
TA–1: CD4+ IL7R+
TA–2: CD4+ PD–1+ TFH/TPH
TA–3: CD4+ IKZF2+ Treg
0 4 1 2 3
RA only T cells
clustering at resolution 0.30
ln(OR)
5.0
2.5
0.0
−2.5
−5.0
b
RA only stromal cells
Stromal chromatin class
SA–1: PRG4+ lining
SA–2: CD34+ MFAP5+ sublining
SA–0: CXCL12+ HLA–DRhi sublining
SA–3: MCAM+ mural
0 3 4 1 2 5
RA only stromal cells
clustering at resolution 0.25
ln(OR)
5
0
−5
−10
c
HCC sample B/plasma cells
B/plasma chromatin class
BA–3: FCER2+ IGHD+ naive B
BA–4: CD24+ MAST4+ unswitched memory B
BA–2: TOX+ PDE4D+ switched memory B
BA–5: ITGAX+ ABC
BA–0: CREB3L2+ plasma
BA–1: CD27+ plasma
3 4 2 6 0 5 1
HCC sample B/plasma cells
clustering at resolution 0.60
ln(OR)
4
0
−4
−8
d
HCC sample endothelial cells
Endothelial chromatin class
EA–2: SEMA3G+ arteriolar
EA–3: PROX1+ lymphatic
EA–0: SELP+ venular
EA–1: RGCC+ capillary
2 3 1 0
HCC sample endothelial cells
clustering at resolution 0.40
ln(OR)
2
1
0
−1
−2

Supplementary Fig. 8. Chromatin classes are stable including or excluding OA and low-cell-count samples.

a. UMAP colored by T cell clusters defined from unimodal scATAC-seq and multimodal snATAC-seq T cells in RA samples only (**left**) and the natural log of the odds ratio between these clusters and the T cell chromatin classes defined in **Fig. 2** (**right**).

b. UMAP colored by stromal clusters defined from unimodal scATAC-seq and multimodal snATAC-seq stromal cells in RA samples only (**left**) and the natural log of the odds ratio between these clusters and the stromal chromatin classes defined in **Fig. 3** (**right**).

c. UMAP colored by B/plasma clusters defined from unimodal scATAC-seq and multimodal snATAC-seq B/plasma cells in high-cell-count (HCC) samples with at least 100 B/plasma cells (**left**) and the natural log of the odds ratio between these clusters and the B/plasma chromatin classes defined in **Fig. 5** (**right**).

d. UMAP colored by endothelial clusters defined from unimodal scATAC-seq and multimodal snATAC-seq endothelial cells in HCC samples with at least 100 endothelial cells (**left**) and the natural log of the odds ratio between these clusters and the endothelial chromatin classes defined in **Fig. 6** (**right**).

In all **right** panels, non-significant (FDR>0.05) OR values are white and the colors of the x-axis labels correspond to the colors in the UMAPs on the **left**.

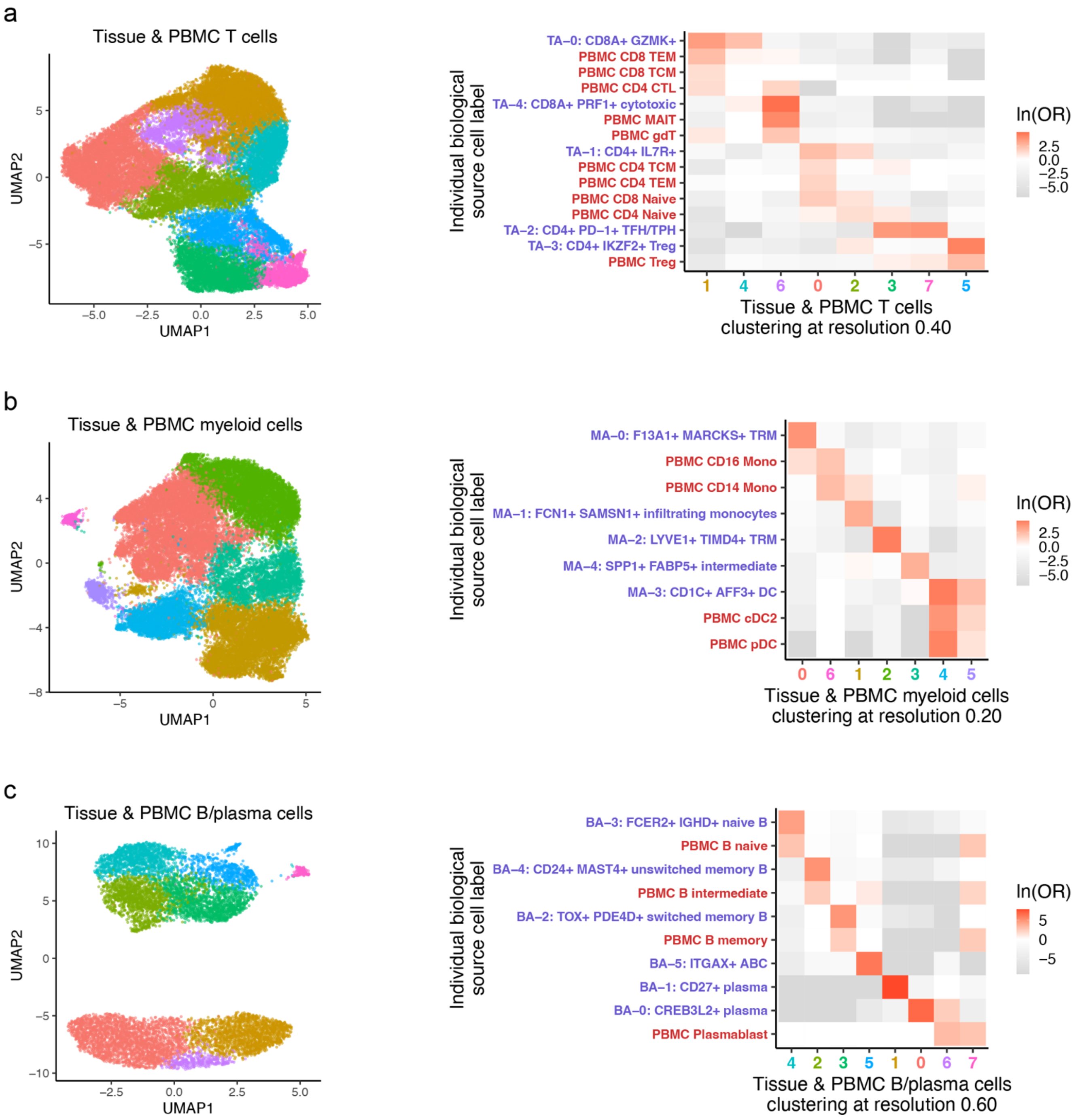

Supplementary Fig. 9. Tissue and PBMC analysis.
Clustering RA unimodal scATAC-seq, RA multimodal snATAC-seq, and healthy PBMC multimodal snATAC-seq together visualized on UMAP (**left**) and the natural log of the odds ratio between these clusters and the tissue/PBMC labels (**right**) for (**a.**) T, (**b.**) myeloid, and (**c.**) B/plasma cells. Non-significant (FDR>0.05) OR values are white. On the **right**, RA tissue chromatin classes are colored in purple and PBMC labels with more than 10 cells are colored in red; the colors of the x-axis labels correspond to the colors in the UMAPs on the **left**.

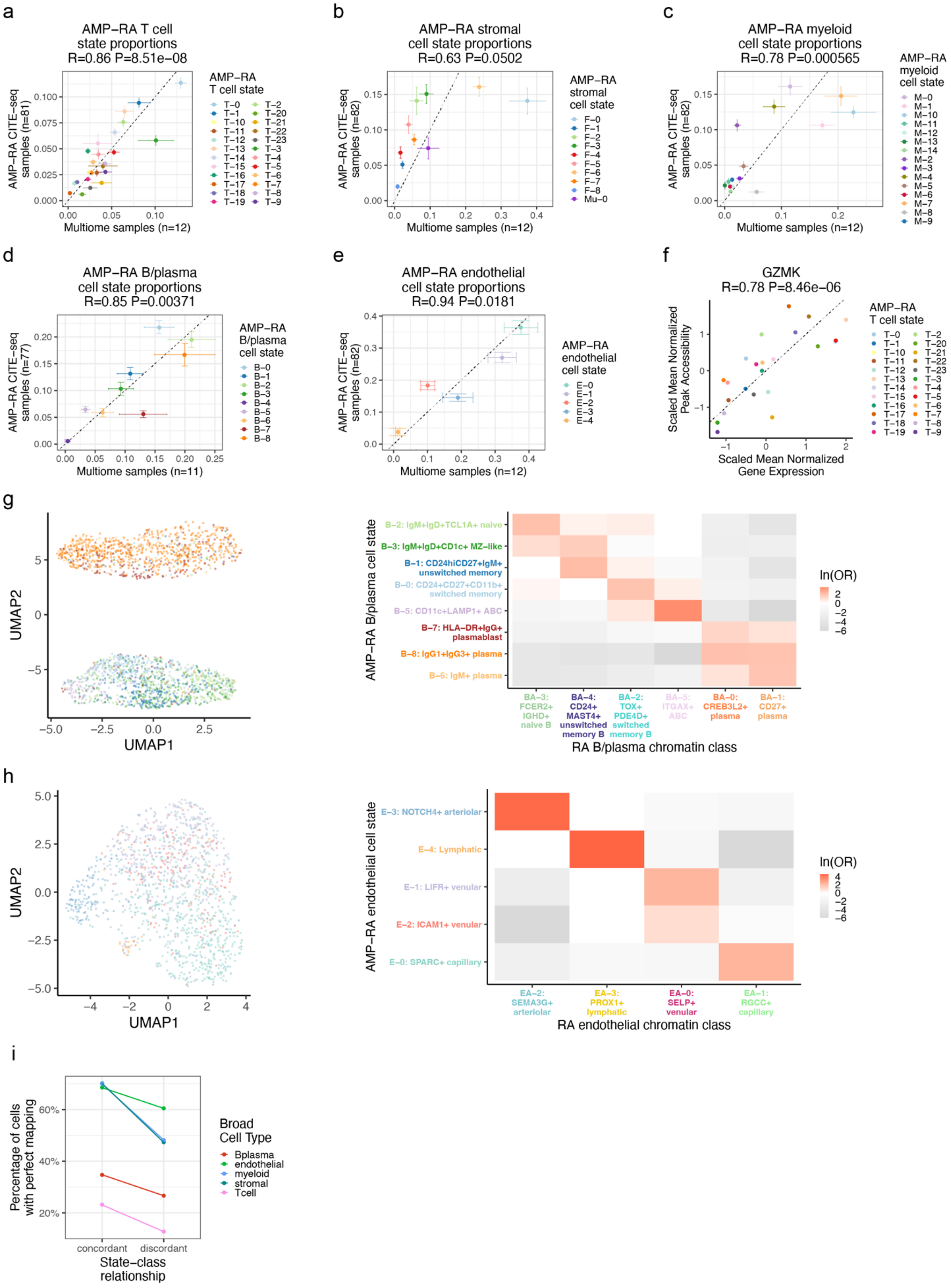

Supplementary Fig. 10. Mapping to transcriptional cell states within the chromatin class superstate model.

a.-e. AMP-RA reference transcriptional cell state proportions across CITE-seq[14] (y-axis) and multiome snRNA-seq (x-axis) samples for (**a.**) T (n=24), (**b.**) stromal (n=10), (**c.**) myeloid (n=15), (**d.**) B/plasma (n=9), and (**e.**) endothelial (n=5) cell states. These cell states were defined originally in the CITE-seq datasets and inferred in the multiome datasets using Symphony[95]. Error bars are standard errors of the means. Pearson correlation coefficients (R) and two-sided p-values (P) also noted.
f. Scaled mean normalized marker gene expression and peak accessibility for *GZMK* across AMP-RA reference transcriptional T cell states in multimodal datasets. Pearson correlation coefficient (R) and two-sided p-value (P) calculated on the 24 T cell states.
g.-h. For (**g.**) B/plasma and (**h.**) endothelial cells, chromatin class UMAP colored by the classified AMP-RA reference transcriptional cell states for multiome cells (**left**) and the natural log of the odds ratio between the chromatin classes and transcriptional cell states (**right**). On the **right**, non-significant values (FDR>0.05) are white, and the colors of the y-axis labels correspond to the colors in the UMAPs on the **left**. In **g.**, the B-4: AICDA+BCL6+ GC-like transcriptional cell state was excluded as fewer than 10 cells were classified into it.
i. For each cell type, the percentage of multiome cells with perfect mapping (*i.e.,* all 5 nearest neighbors in the reference had the same cell state) segregated by whether the cell's classified transcriptional cell state was in the corresponding chromatin class ('concordant') or not ('discordant'), as determined by the OR (**Fig. 7a-c**, **Supplementary Fig. 10g-h**).

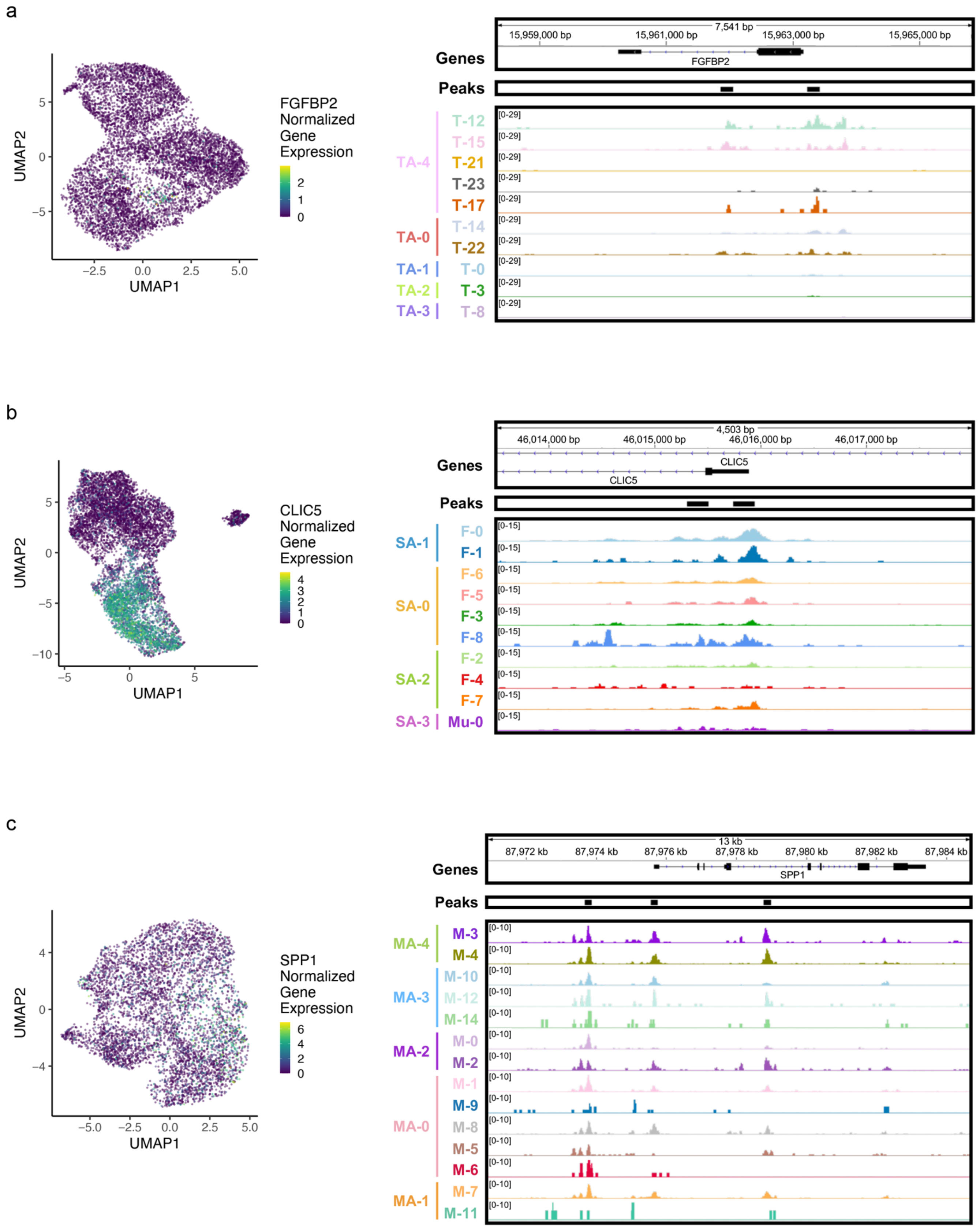

Supplementary Fig. 11. Open chromatin reads aggregated by transcriptional cell states and visualized at gene loci.

Chromatin class UMAP colored by normalized gene expression of multiome cells (**left**) and gene loci with selected gene isoforms, open chromatin peaks, and chromatin accessibility reads aggregated by transcriptional cell state and scaled by cell counts per state (**Methods**) (**right**) for (**a.**) *FGFBP2* (chr4:15,958,313-15,965,852) in T cells, (**b.**) *CLIC5* (chr6:46,013,500-46,018,000) in stromal cells, and (**c.**) *SPP1* (chr4:87,970,884-87,984,690) in myeloid cells. The associated chromatin classes were also listed, though not all states therein were shown. In **c.**, M-13: pDC was excluded in myeloid cells since less than 10 multiome cells were classified as such.

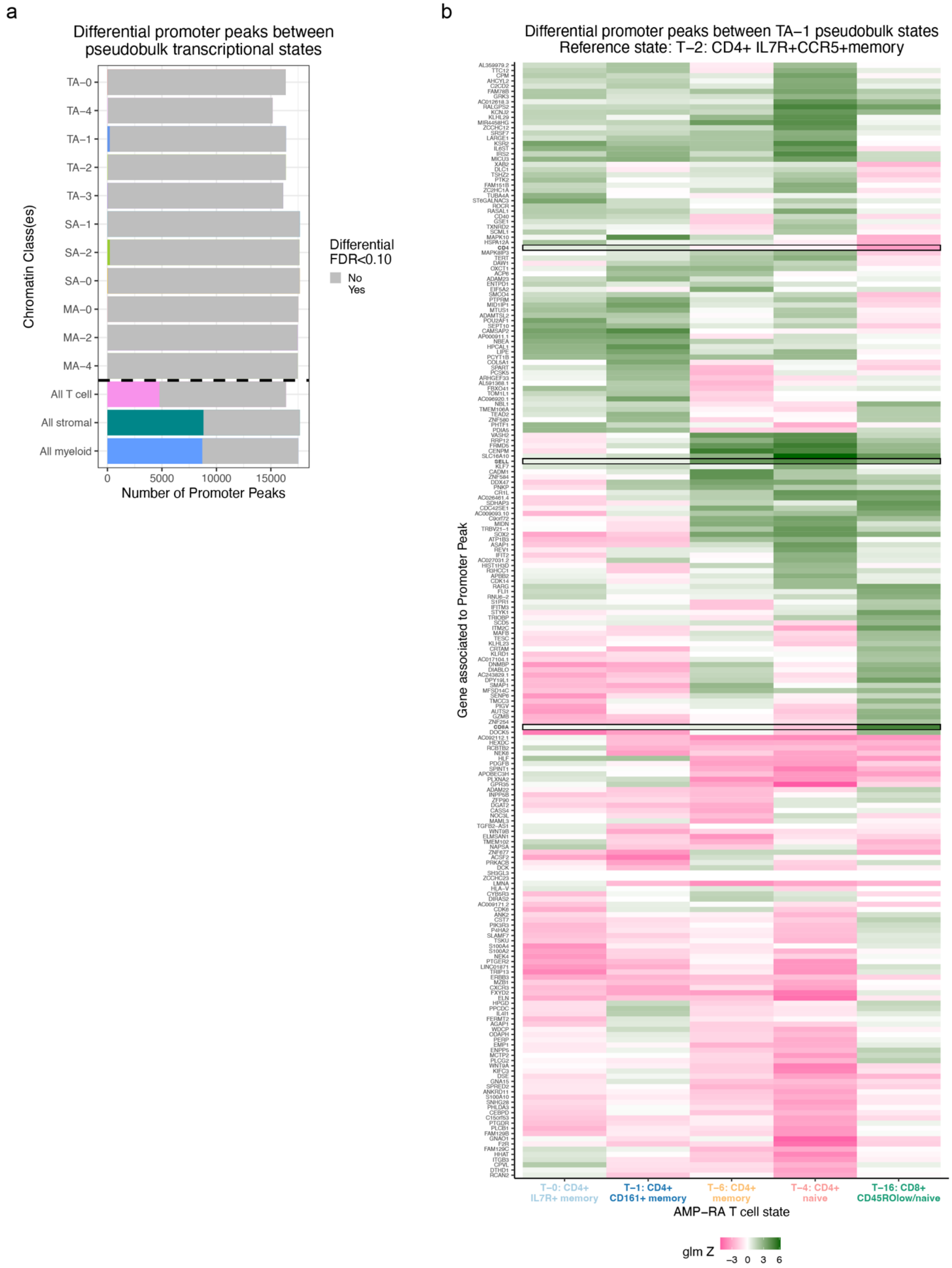

Supplementary Fig. 12. Differential promoter peaks between pseudobulks by sample and transcriptional cell states within or across chromatin classes.

a. The number of differential promoter peaks between pseudobulked transcriptional cell states within or across classes, as determined by an ANOVA LRT FDR<0.10 between two negative binomial models (**Methods**), colored by class or cell type; peaks with FDR≥0.10 are colored in gray. A dashed line separates the within and across class analyses. Pseudobulks were calculated by summing the non-binary peaks x cells matrix by sample and transcriptional cell state combinations across cells.
b. For the T_A-1: CD4+ IL7R+ chromatin class, the glm Z-score of the pseudobulked transcriptional cell state beta for each differential peak, labeled by the gene associated with that promoter (**Methods**). T-2: CD4+ IL7R+ CCR5+ memory was used as the reference cell type in the negative binomial model. We highlighted promoter peaks associated to *CD4*, *CD8A*, and *SELL* with bolded gene names and a black box outline.

a

b

c

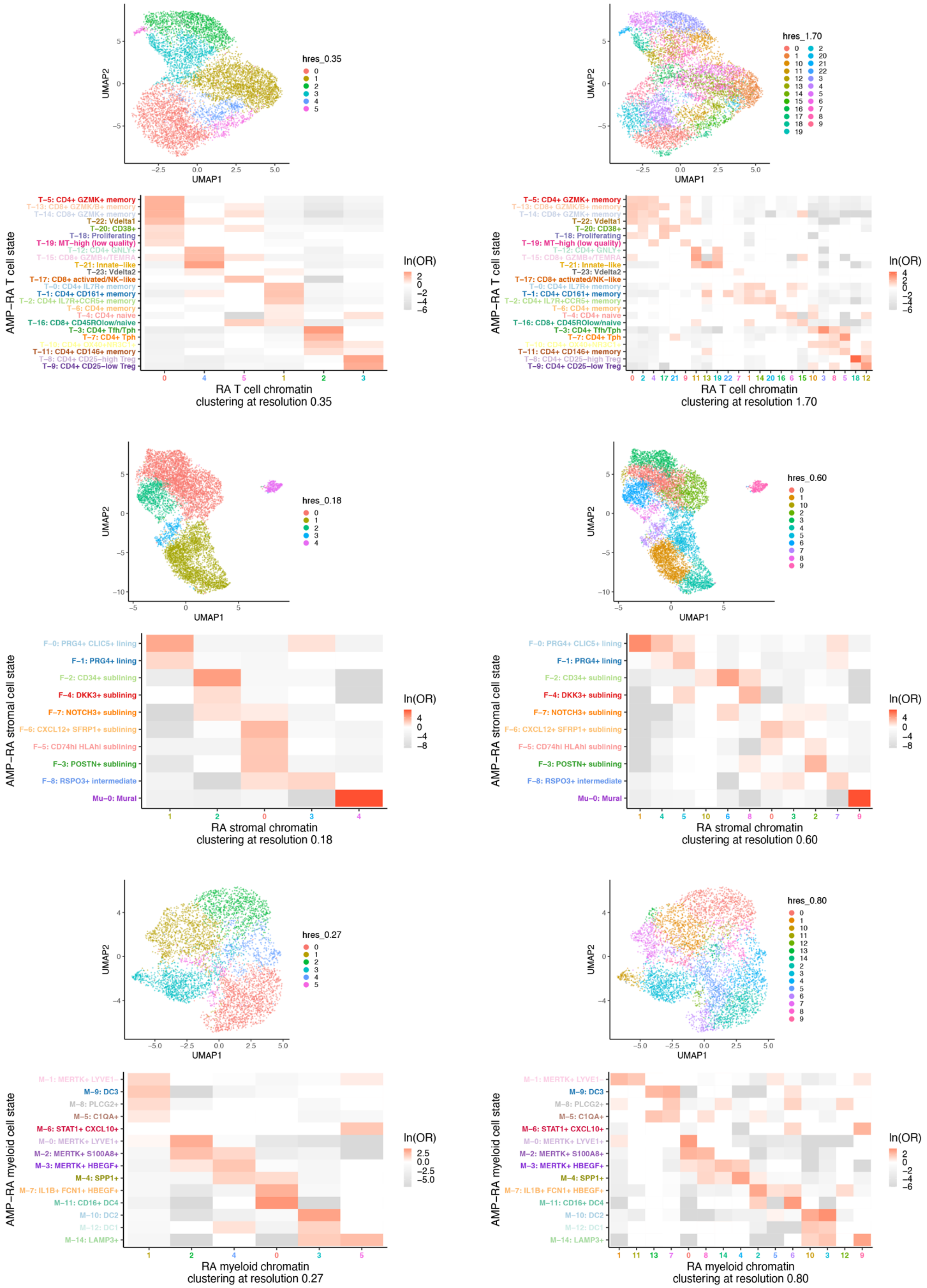

hres_0.35
hres_1.70
UMAP1
UMAP2
T-5: CD4+ GZMK+ memory
T-13: CD8+ GZMK/B+ memory
T-14: CD8+ GZMK+ memory
T-22: Vdelta1
T-20: CD38+
T-18: Proliferating
T-19: MT-high (low quality)
T-12: CD4+ GNLY+
T-15: CD8+ GZMB+/TEMRA
T-21: Innate-like
T-23: Vdelta2
T-17: CD8+ activated/NK-like
T-0: CD4+ IL7R+ memory
T-1: CD4+ CD161+ memory
T-2: CD4+ IL7R+CCR5+ memory
T-6: CD4+ memory
T-4: CD4+ naive
T-16: CD8+ CD45ROlow/naive
T-3: CD4+ Tfh/Tph
T-7: CD4+ Tph
T-11: CD4+ CD146+ memory
T-8: CD4+ CD25-high Treg
T-9: CD4+ CD25-low Treg
AMP-RA T cell state
ln(OR)
RA T cell chromatin clustering at resolution 0.35
RA T cell chromatin clustering at resolution 1.70
hres_0.18
hres_0.60
F-0: PRG4+ CLIC5+ lining
F-1: PRG4+ lining
F-2: CD34+ sublining
F-4: DKK3+ sublining
F-7: NOTCH3+ sublining
F-6: CXCL12+ SFRP1+ sublining
F-5: CD74hi HLAhi sublining
F-3: POSTN+ sublining
F-8: RSPO3+ intermediate
Mu-0: Mural
AMP-RA stromal cell state
RA stromal chromatin clustering at resolution 0.18
RA stromal chromatin clustering at resolution 0.60
hres_0.27
hres_0.80
M-1: MERTK+ LYVE1-
M-9: DC3
M-8: PLCG2+
M-5: C1QA+
M-6: STAT1+ CXCL10+
M-0: MERTK+ LYVE1+
M-2: MERTK+ S100A8+
M-3: MERTK+ HBEGF+
M-4: SPP1+
M-7: IL1B+ FCN1+ HBEGF+
M-11: CD16+ DC4
M-10: DC2
M-12: DC1
M-14: LAMP3+
AMP-RA myeloid cell state
RA myeloid chromatin clustering at resolution 0.27
RA myeloid chromatin clustering at resolution 0.80

Supplementary Fig. 13. Additional chromatin clustering resolutions.
Chromatin class UMAP colored by chromatin clusters (**top**) and the natural log of the odds ratio between the chromatin clusters and transcriptional cell states (**bottom**) at clustering resolutions that add 1 additional cluster (**left**) and roughly the same number of clusters as states (**right**) for (**a.**) T, (**b.**) stromal, and (**c.**) myeloid cell types. In all **bottom** panels, non-significant (FDR>0.05) OR values are white and the colors of the x-axis labels correspond to the colors in the UMAPs on the **top**.

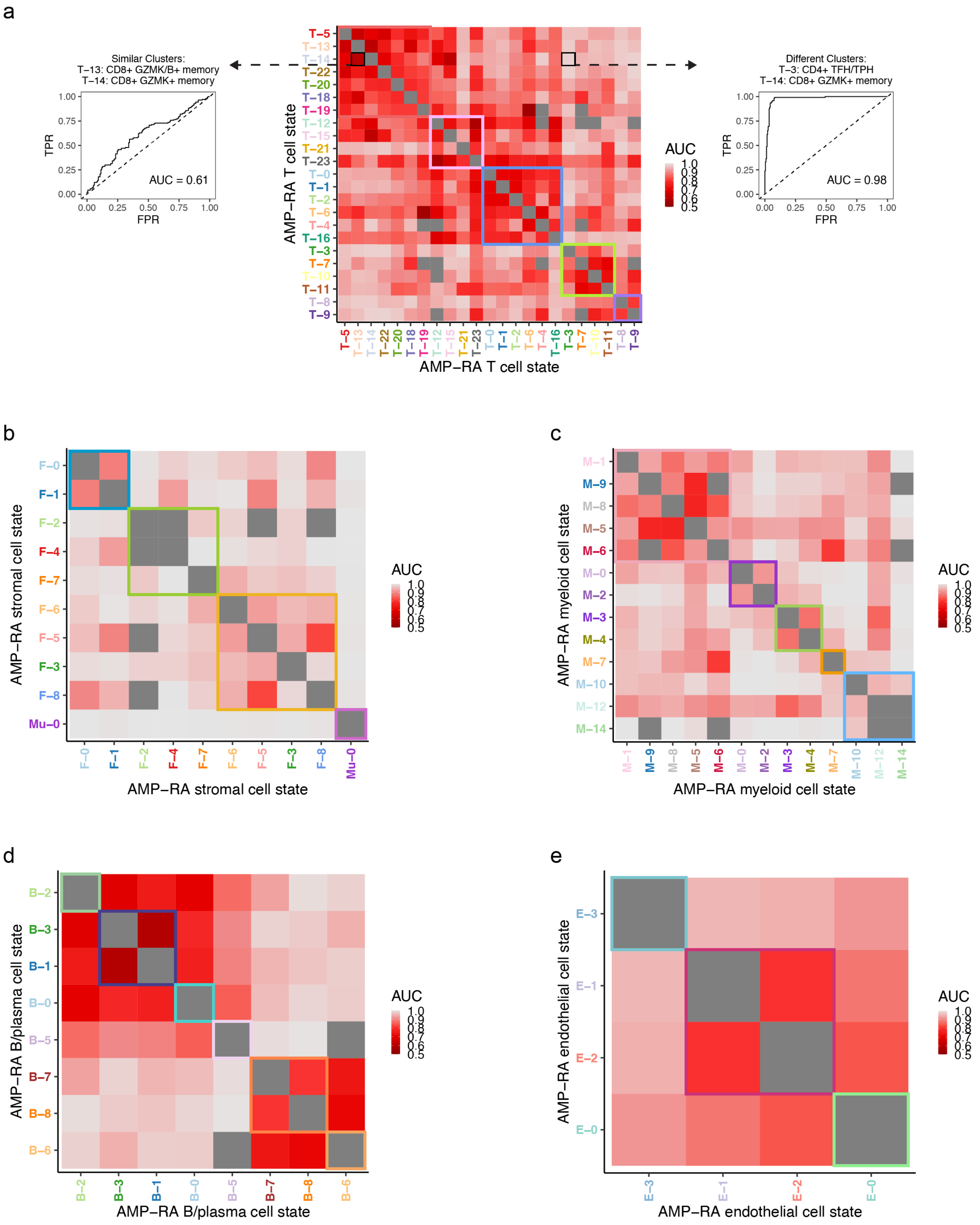

Supplementary Fig. 14. Linear Discriminant Analysis (LDA) model predicted pairwise transcriptional cell state from continuous chromatin principal components (PCs).

a. Linear Discriminant Analysis (LDA) model predicted the transcriptional cell state for each pair of T cell states from the batch-corrected chromatin principal components (PCs) (**Methods**). Area under the curve (AUC) values shown for an example of similar states (**left**) and different states (**right**) with all pairs of states with more than 50 cells shown in the **middle**. Upper and lower triangles are mirrored; the LDA analysis was run only once per pair of states. The grey non-diagonal values depict pairs of states that had a constant variable in the model due to samples with too few cells for that pair. The superimposed colored boxes reflect chromatin class superstates defined in **Fig. 7a-c** and **Supplementary Fig. 10g-h**.
b.-e. Middle panel of (**a.**) for (**b.**) stromal, (**c.**) myeloid, (**d.**) B/plasma, and (**e.**) endothelial cell types.

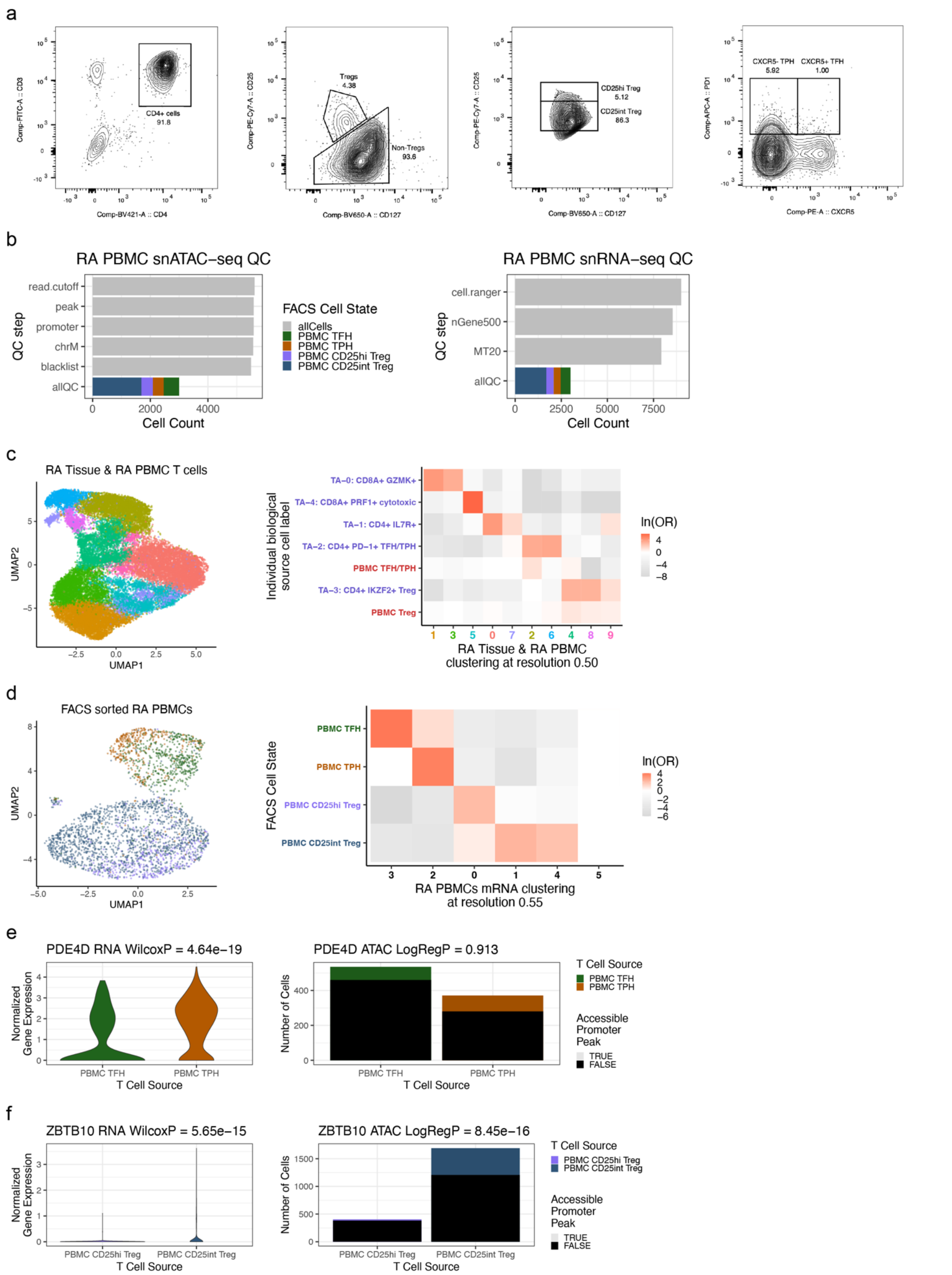

Supplementary Fig. 15. Multiome experimental support for the hypothesized superstate model using RA PBMCs sorted for Treg, TFH, TPH populations via FACS.

a. FACS plots of pooled PBMCs from 4 RA patients sorted for: $CD4^+CD127^-CD25^{hi}$ Treg, $CD4^+CD127^-CD25^{int}$ Treg, $CD4^+CD25^-PD1^+CXCR5^+$ TFH, and $CD4^+CD25^-PD1^+CXCR5^-$ TPH.
b. Quality control steps ending in the final cell counts for the FACS cell state hashtags in snATAC-seq (**left**) and snRNA-seq (**right**). allQC refers to cells passing ATAC, RNA, and HTO quality control measures (**Methods**).
c. Clustering RA tissue unimodal scATAC-seq, RA tissue multimodal snATAC-seq, and sorted RA PBMC multimodal snATAC-seq cells together visualized on UMAP (**left**) and the natural log of the odds ratio between these clusters and the RA tissue/RA PBMC labels (**right**). Non-significant (FDR>0.05) OR values are white. The colors of the x-axis labels on the **right** correspond to the colors in the UMAPs on the **left**. On the **right** y-axis, RA tissue chromatin classes are colored in purple and RA PBMC FACS cell states are colored in red.
d. RA PBMC multimodal snRNA-seq cells visualized on UMAP (**left**) and the natural log of the odds ratio between the snRNA-seq clusters and the RA PBMC FACS cell states (**right**). Non-significant (FDR>0.05) OR values are white. The colors of the y-axis labels on the **right** correspond to the RA PBMC FACS cell states in the UMAPs on the **left**.
e.-f. Normalized gene expression (**left**) and proportion of cells with or without chromatin accessibility in the promoter peak (**right**) for (**e.**) *PDE4D*, segregated by RA PBMC TFH and TPH populations and (**f.**) *ZBTB10*, segregated by RA PBMC $CD25^{hi}$ and $CD25^{int}$ Treg populations. Nominal two-sided Wilcoxon (**left**) and logistic regression LRT (**right**) P-values above (**Methods**).

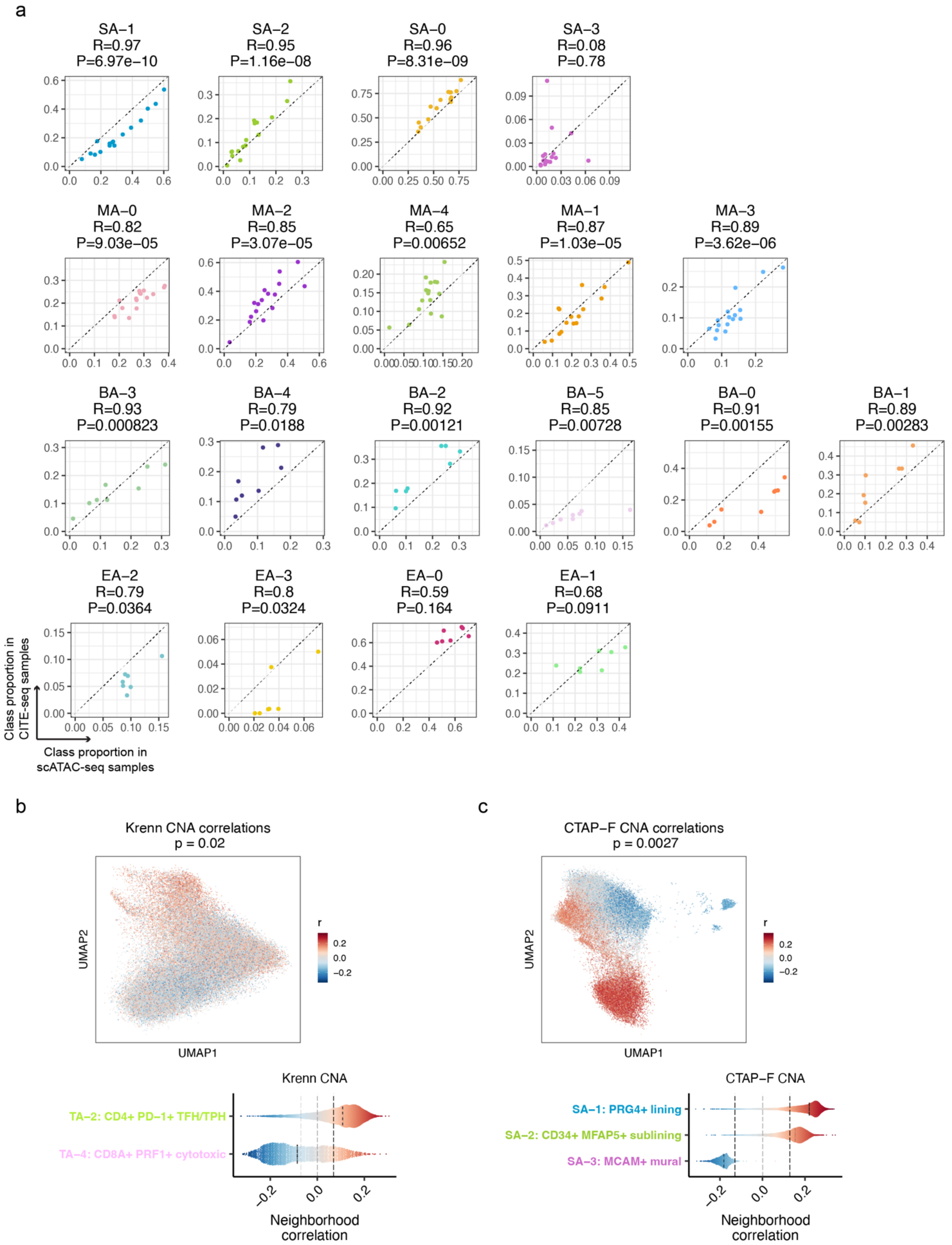

Supplementary Fig. 16. Additional RA CNA correlations.
a. For each donor shared between the unimodal scATAC-seq and AMP-RA reference studies with at

least 200 cells for that cell type (or 100 cells for endothelial cells), the Pearson correlation (R) and two-sided p-value (P) between the relative proportions of chromatin classes defined in the unimodal scATAC-seq datasets (x-axis) and classified into in the CITE-seq datasets[14] through the multiome cells (y-axis). Number of donors: 16 in stromal, 16 in myeloid, 8 in B/plasma, 7 in endothelial.
b. CNA correlations between T cell neighborhoods and Krenn inflammation score in AMP-RA reference T cells visualized on chromatin class UMAP (**top**; two-sided global P=0.02) and aggregated by classified T cell chromatin classes (**bottom**). On the **top**, cells not passing the FDR threshold were colored grey. On the **bottom**, FDR thresholds shown in dotted black lines.
c. CNA correlations between stromal cell neighborhoods and CTAP-F in AMP-RA reference stromal cells visualized on chromatin class UMAP (**top**; two-sided global P=0.0027) and aggregated by classified stromal chromatin classes (**bottom**). On the **top**, cells not passing the FDR threshold were colored grey. On the **bottom**, FDR thresholds shown in dotted black lines.

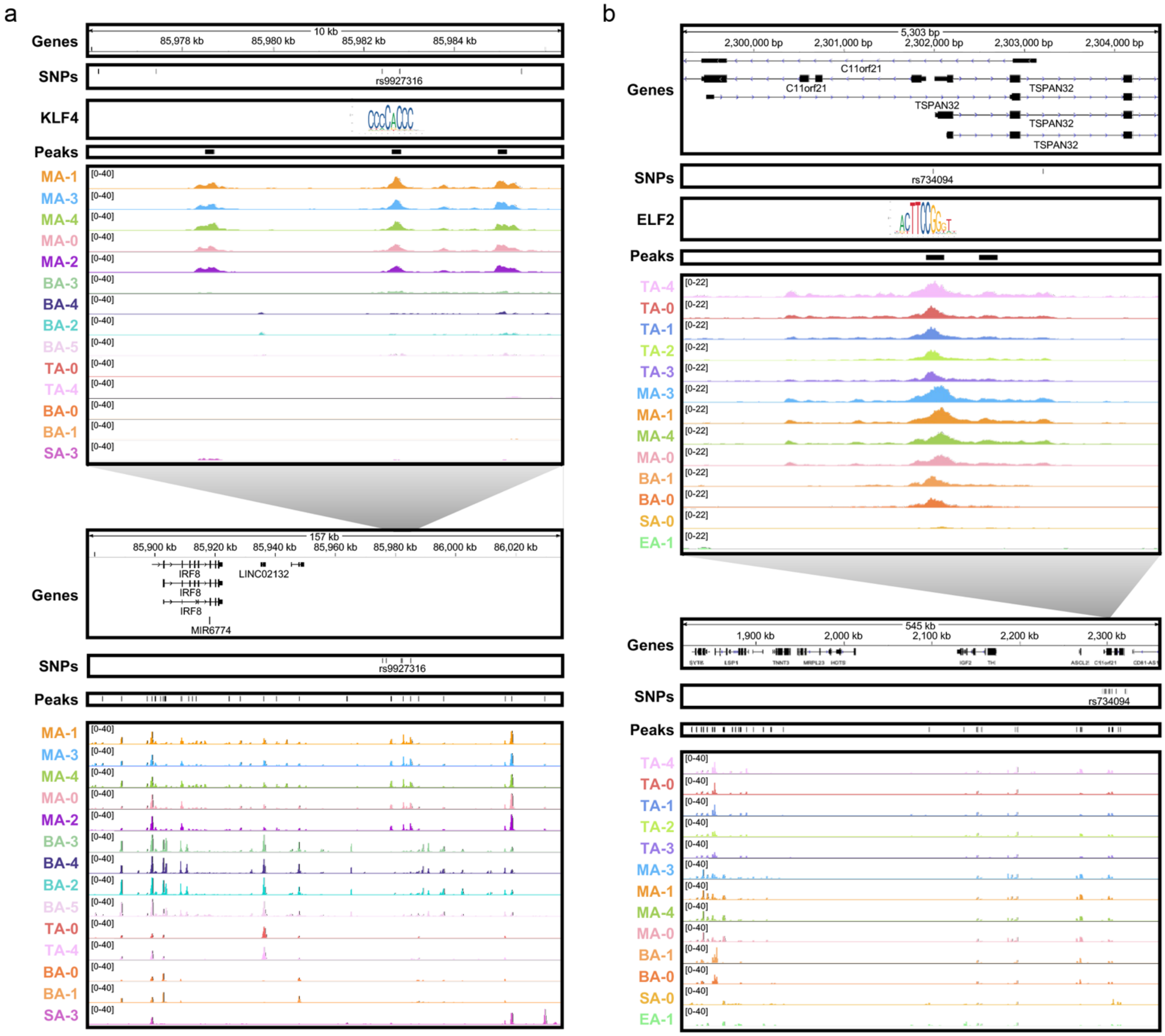

Supplementary Fig. 17. Chromatin accessibility of additional RA risk variants aggregated by chromatin classes and visualized at gene loci.

a. rs9927316 locus, zoomed in (chr16:85,975,899-85,986,400) (**top**) and zoomed out (chr16:85,877,695-86,035,055) (**bottom**) with gene isoforms, SNPs, open chromatin peaks, and chromatin accessibility reads aggregated by chromatin class and scaled by read counts per class (**Methods**). KLF4 motif was downloaded from JASPAR[109] ID MA0039.4 and is not to scale, but it is aligned to the SNP-breaking motif position.

b. rs734094, zoomed in (chr11:2,299,199-2,304,500) (**top**) and zoomed out (chr11:1,815,000-2,360,000) (**bottom**) with gene isoforms, SNPs, open chromatin peaks, and chromatin accessibility reads aggregated by chromatin class and scaled by read counts per class (**Methods**). ELF2 motif was downloaded from JASPAR[109] ID MA1483.1 and is not to scale, but it is aligned to the SNP-breaking motif position.

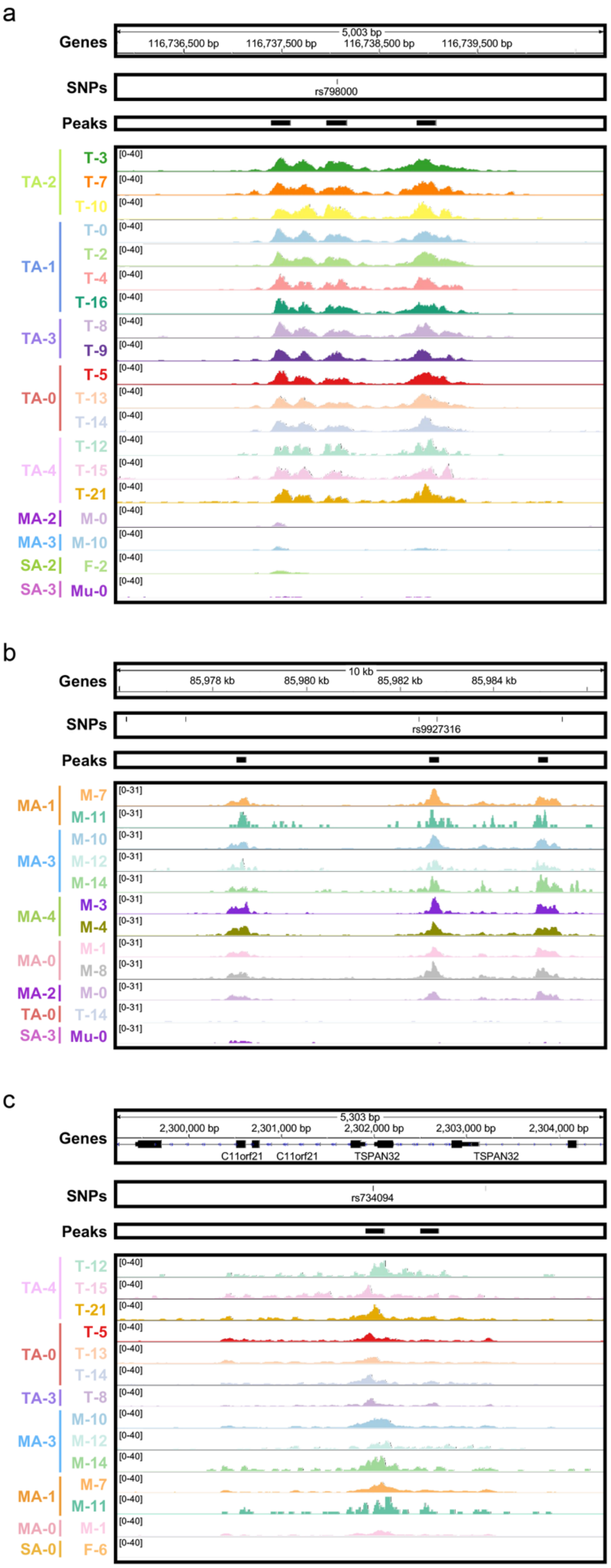

Supplementary Fig. 18. Chromatin accessibility of putative RA risk variants aggregated by transcriptional cell states and visualized at gene loci.

Gene isoforms, SNPs, open chromatin peaks, and chromatin accessibility reads from multiome cells aggregated by transcriptional cell state and scaled by read counts per state (**Methods**) for (**a.**) rs798000 locus (chr1:116,735,799-116,740,800), (**b.**) rs9927316 locus (chr16:85,975,899-85,986,400), and (**c.**) rs734094 locus (chr11:2,299,199-2,304,500). The associated chromatin classes were also listed, though not all states therein were shown.

Supplementary Table 1. Tissue datasets used in this study. BWH: Brigham and Women's Hospital, Boston, MA. HSS: Hospital for Special Surgery Research Institute, New York, NY

Experiment Type	Number of samples	Average Age (years)	Number of Females	Number of CCP positive	Number of CCP negative	RA	OA	Sites	ATAC	RNA	Surface Protein
scATAC-seq	18	65	17	13	1	14	4	7 AMP-RA sites	X		
Multiome	12	60	10	5	4	11	1	BWH & HSS	X	X	
CITE-seq	82	57	59	54	13	73	9	7 AMP-RA sites		X	X

Supplementary Table 2. Mean quality control metrics for ATAC cells segregated by ATAC assay and cell type. See ATAC cell QC **Methods** section for more information.

ATAC Assay	Cell Type	Pre-duplication read counts	Deduplicated read counts	Peak neighborhood read counts	Promoter read counts	Mitochondrial read counts	Blacklisted region read counts
unimodal	Bplasma	38,620	24,476	18,741	8,131	829	1,533
unimodal	endothelial	51,628	31,231	21,040	8,326	1,481	1,775
unimodal	myeloid	38,241	25,243	19,533	6,927	345	1,079
unimodal	NK	41,180	26,042	21,002	9,194	576	1,249
unimodal	stromal	48,412	30,396	20,299	6,943	746	1,843
unimodal	Tcell	40,580	25,079	19,955	8,932	824	1,401
multimodal	Bplasma	124,867	46,437	36,592	15,939	1,336	5,021
multimodal	endothelial	109,231	45,591	31,673	12,514	1,465	4,085
multimodal	myeloid	139,873	58,182	44,318	15,461	1,026	4,085
multimodal	NK	137,460	48,150	38,121	16,850	1,265	4,554
multimodal	stromal	142,374	61,606	42,097	14,588	754	5,811
multimodal	Tcell	131,795	44,828	36,082	16,450	1,264	4,666

Supplementary Table 3. CD4/CD8 promoter peak accessibility in cells across T cell chromatin classes. A plus sign (+) signifies that the CD4 or CD8 lineage promoter peak is accessible while a minus sign (-) signifies that it is not.

Cell counts	Lineage promoter peak status		
Class	CD8A+ CD4-	double positive or negative	CD8A- CD4+
TA-0	1526	5763	84
TA-1	391	5584	593
TA-2	120	3645	601
TA-3	35	2847	211
TA-4	319	1421	28

Supplementary Table 4. The absolute cell counts and relative frequencies of OA and RA cells in each chromatin class.

Chromatin Classes	Cell Counts		Cell Percentages	
	OA	RA	OA	RA
TA-0: CD8A+ GZMK+	637	6736	9%	91%
TA-1: CD4+ IL7R+	805	5763	12%	88%
TA-2: CD4+ PD-1+ TFH/TPH	432	3934	10%	90%
TA-3: CD4+ IKZF2+ Treg	357	2736	12%	88%
TA-4: CD8A+ PRF1+ cytotoxic	164	1604	9%	91%
SA-0: CXCL12+ HLA-DRhi sublining	1812	9921	15%	85%
SA-1: PRG4+ lining	2210	7197	23%	77%
SA-2: CD34+ MFAP5+ sublining	371	2261	14%	86%
SA-3: MCAM+ mural	69	466	13%	87%
MA-0: F13A1+ MARCKS+ TRM	1251	5530	18%	82%
MA-1: FCN1+ SAMSN1+ infiltrating monocytes	725	5460	12%	88%
MA-2: LYVE1+ TIMD4+ TRM	1140	4866	19%	81%
MA-3: CD1C+ AFF3+ DC	762	3028	20%	80%
MA-4: SPP1+ FABP5+ intermediate	303	2626	10%	90%
BA-0: CREB3L2+ plasma	118	2752	4%	96%
BA-1: CD27+ plasma	61	1590	4%	96%
BA-2: TOX+ PDE4D+ switched memory B	246	1201	17%	83%
BA-3: FCER2+ IGHD+ naive B	237	1053	18%	82%
BA-4: CD24+ MAST4+ unswitched memory B	236	636	27%	73%
BA-5: ITGAX+ ABC	116	395	23%	77%
EA-0: SELP+ venular	238	2146	10%	90%
EA-1: RGCC+ capillary	55	893	6%	94%
EA-2: SEMA3G+ arteriolar	30	305	9%	91%
EA-3: PROX1+ lymphatic	13	129	9%	91%

Supplementary Table 5. RA risk variants with PIP>0.1 from Ishigaki, Sakaue, Terao, et al., Nat Genet, 2022 that overlap our trimmed peaks. SCENT genes are the SNP's best guess target gene from our sister study (Sakaue et al., Nat Genet, 2024). TF motifs from ArchR's JASPAR2020 motif set (Granja et al., Nat Genet., 2021; Castro-Mondragon, NAR, 2022). Peak class accessibility determined by scaled mean normalized peak accessibility over 24 classes and 11 peaks, z > 1.

SNP rsID	SNP chr	SNP start	SNP stop	SNP PIP	SCENT gene	SNP overlapping TF motif	Peak chr	Peak start	Peak stop	Peak class accessibility
rs11209051	chr1	67333211	67333212	0.1388	IL12RB2, SERBP1	NA	chr1	67333106	67333306	TA-0, TA-4, TA-2, TA-3
rs798000	chr1	116738073	116738074	0.9999	CD2	IRF1, STAT1/2,MEF2A, MEF2C, ZNF384	chr1	116737968	116738168	TA-0, TA-1, TA-2, TA-3
rs734094	chr11	2301989	2301990	0.4082	LSP1	ZBTB7A, ELF2	chr11	2301916	2302116	TA-4, MA-1, MA-3
rs911760	chr9	5438434	5438435	0.2241	NA	NA	chr9	5438284	5438484	TA-4, TA-1, BA-4, BA-5, BA-1
rs663743	chr11	64340262	64340263	0.2075	NA	ESR1	chr11	64340084	64340284	BA-3, BA-4, BA-2, BA-5
rs3784099	chr14	68283209	68283210	0.1061	RAD51B	ZNF382, HOXA10, HOXB13, HOXD3, CDX1, CDX2, CDX4	chr14	68283072	68283272	TA-1, TA-2, BA-3, BA-4, BA-2
rs4840568	chr8	11493509	11493510	0.1159	CTSB	NA	chr8	11493501	11493701	BA-3, BA-4, BA-2, BA-5
rs9927316	chr16	85982794	85982795	0.5360	NA	KLF2, KLF4, KLF5, KLF15, ZNF740	chr16	85982638	85982838	MA-0, MA-2, MA-4, MA-1, MA-3
rs72798636	chr5	134506252	134506253	0.1257	JADE2, PCBD2	HIC2	chr5	134506244	134506444	EA-2, EA-0, EA-1, SA-1
rs71508903	chr10	62020111	62020112	0.1255	NA	NA	chr10	62020060	62020260	EA-2, EA-3, EA-0, EA-1
rs7441808	chr4	26088752	26088753	0.1404	RBPJ	NA	chr4	26088619	26088819	TA-2, SA-2

Supplementary Table 6. Selected main results per chromatin class: TF motifs, transcriptional cell states, RA CNA associations, and RA risk variants. In column "RA CNA associations," clinical metrics and cell type abundance phenotypes (CTAPs) were listed if the AMP-RA reference cells within their Symphony-classified chromatin class were significantly expanded (+) or depleted (-) in association with that patient attribute (**Methods**).

Cell type	Chromatin class	TF motifs	Corresponding transcriptional cell states	RA CNA associations	RA risk variants
T cells	TA-0: CD8A+ GZMK+	EOMES<br>TBX21<br>IRF4	T-5: CD4+ GZMK+ memory<br>T-13: CD8+ GZMK/B+ memory<br>T-14: CD8+ GZMK+ memory<br>T-22: Vdelta1		rs11209051<br>rs798000
	TA-4: CD8A+ PRF1+ cytotoxic	EOMES<br>TBX21<br>RUNX3	T-12: CD4+ GNLY<br>T-15: CD8+ GZMB+/TEMRA<br>T-21: Innate-like<br>T-23: Vdelta2	Lymphoid Aggregates (-)<br>Krenn (-)<br>CTAP-TB (-)	rs11209051<br>rs734094<br>rs911760
	TA-1: CD4+ IL7R+	TCF7<br>LEF1<br>GATA3	T-0: CD4+ IL7R+ memory<br>T-1: CD4+ CD161+ memory<br>T-4: CD4+ naive<br>T-16: CD8+ CD45ROlow/naive		rs798000<br>rs911760<br>rs3784099
	TA-2: CD4+ PD-1+ TFH/TPH	BATF<br>JUN<br>FOS	T-3: CD4+ Tfh/Tph<br>T-7: CD4+ Tph<br>T-10: CD4+ OX40+ NR3C1+<br>T-11: CD4+ CD146+ memory	Krenn (+)<br>CTAP-TB (+)	rs11209051<br>rs798000<br>rs3784099<br>rs7441808
	TA-3: CD4+ IKZF2+ Treg	KLF10<br>SP3	T-8: CD4+ CD25-high Treg<br>T-9: CD4+ CD25-low Treg		rs11209051<br>rs798000
Stromal cells	SA-1: PRG4+ lining	FOS<br>JUND<br>BATF	F-0: PRG4+ CLIC5+ lining<br>F-1: PRG4+ lining	CTAP-F (+)<br>CTAP-M (-)	rs72798636
	SA-2: CD34+ MFAP5+ sublining	NFATC4<br>NFATC3	F-2: CD34+ sublining<br>F-4: DKK3+ sublining<br>F-7: NOTCH3+ sublining	CTAP-F (+)	rs7441808
	SA-0: CXCL12+ HLA-DRhi sublining	TEAD1<br>STAT1<br>STAT3	F-6: CXCL12+ SFRP1+ sublining<br>F-5: CD74hi HLAhi sublining<br>F-3: POSTN+ sublining<br>F-8: RSPO3+ intermediate		
	SA-3: MCAM+ mural	KLF2<br>EBF1	Mu-0: Mural	CTAP-F (-)<br>CTAP-M (+)<br>CTAP-TF (-)	
Myeloid cells	MA-0: F13A1+ MARCKS+ TRM	RELA<br>SREBF2	M-1: MERTK+ LYVE1-<br>M-9: DC3<br>M-8: PLCG2+<br>M-5: C1QA+<br>M-6: STAT1+ CXCL10+		rs9927316
	MA-2: LYVE1+ TIMD4+ TRM	KLF4<br>NFIX<br>NFATC3	M-0: MERTK+ LYVE1+<br>M-2: MERTK+ S100A8+	Lymphoid Density (-)<br>CTAP-EFM (+)<br>CTAP-F (+)	rs9927316
	MA-4: SPP1+ FABP5+ intermediate	JUN<br>FOS	M-3: MERTK+ HBEGF+<br>M-4: SPP1+	Lymphoid Density (+)<br>Lymphoid Aggregates (+)<br>Krenn (+)<br>CTAP-M (+)	rs9927316
	MA-1: FCN1+ SAMSN1+ infiltrating monocytes	JUN<br>FOSL1<br>CEBPD	M-7: IL1B+ FCN1+ HBEGF+<br>M-11: CD16+ DC4	Lymphoid Aggregates (-)<br>CTAP-EFM (-)	rs9927316<br>rs734094
	MA-3: CD1C+ AFF3+ DC	SPI1<br>IRF1<br>STAT1/2	M-10: DC2<br>M-12: DC1<br>M-14: LAMP3+		rs9927316<br>rs734094
B/plasma cells	BA-3: FCER2+ IGHD+ naive B	SPIB<br>SPI1	B-2: IgM+IgD+TCL1A+ naive	Lymphoid Aggregates (-)	rs663743<br>rs3784099<br>rs4840568
	BA-4: CD24+ MAST4+ unswitched memory B	NFKB1<br>ZBTB7A	B-3: IgM+IgD+CD1c+ MZ-like<br>B-1: CD24hiCD27+IgM+ unswitched memory		rs911760<br>rs663743<br>rs3784099<br>rs4840568
	BA-2: TOX+ PDE4D+ switched memory B	ETS1<br>RELA	B-0: CD24+CD27+CD11b+ switched memory	CTAP-TB (+)	rs663743<br>rs3784099<br>rs4840568
	BA-5: ITGAX+ ABC	TBX19	B-5: CD11c+LAMP1+ ABC		rs911760<br>rs663743<br>rs4840568
	BA-0: CREB3L2+ plasma	KLF2<br>SP3	B-7: HLA-DR+IgG+ plasmablast<br>B-8: IgG1+IgG3+ plasma	CTAP-TB (-)	
	BA-1: CD27+ plasma	BATF<br>JUN	B-6: IgM+ plasma	CTAP-TB (-)	rs911760
Endothelial cells	EA-2: SEMA3G+ arteriolar	SOX17	E-3: NOTCH4+ arteriolar		rs71508903<br>rs72798636
	EA-3: PROX1+ lymphatic		E-4: Lymphatic	CTAP-F (+)<br>CTAP-M (-)	rs71508903
	EA-0: SELP+ venular	STAT3<br>NFIB	E-1: LIFR+ venular<br>E-2: ICAM1+ venular	CTAP-F (+)	rs71508903<br>rs72798636
	EA-1: RGCC+ capillary	FOS<br>JUN	E-0: SPARC+ capillary	CTAP-F (-)<br>CTAP-M (+)	rs71508903<br>rs72798636
