# Supplementary material for: The chromatin landscape of pathogenic transcriptional cell states in rheumatoid arthritis
Source: Nat Commun. 2024 May 31;15:4650. doi: 10.1038/s41467-024-48620-7 (PMC11143375; doi:10.1038/s41467-024-48620-7)
Supplement: Supplementary file 4 — Description of additional supplementary files [file 41467_2024_48620_MOESM4_ESM.docx]

**Description of additional supplementary files**

**Supplementary Data 1.** Logistic regression model relating promoter peak openness to CD4/CD8A promoter peak accessibility status (1 if CD4+ CD8A-; -1 if CD4- CD8A+; 0 otherwise). A plus sign (+) signifies that the CD4 or CD8 lineage promoter peak is accessible while a minus sign (-) signifies that it is not. We controlled for chromatin class, donor, and read depth. A positive lineage beta depicted CD4 association while a negative lineage beta showed CD8A association. Results with Likelihood Ratio Test (LRT) FDR < 0.2 shown.

**Supplementary Data 2.** One-sided Wilcoxon test between the normalized gene expression of the TF gene in cells in the specified chromatin class ('In Class') and all other cells in that cell type ('Outside of Class'). The TFs chosen correspond to the top motifs enriched in class-specific accessible chromatin from **Figs. 2-6c, right**. FDRs calculated within cell types.

**Supplementary Data 3.** Odds ratios (ORs) between RA chromatin class and transcriptional cell state membership. All statistics are calculated within cell types. Multiple hypothesis test correction via FDR.

**Supplementary Data 4.** Markers for each chromatin class. The top 5 peaks ('T5P') from a logistic regression model relating class to binary peak distributions over all unimodal and multimodal cells (**Methods**), with at least log2FC>0.5 and a log-likelihood ratio test -log10(FDR)>5, ordered by FDR, are shown. The top 5 genes ('T5G') from a two-sided Wilcoxon test via presto relating class to normalized gene expression over multiome cells (**Methods**), with at least logFC>0.5 and an -log10(FDR)>5, ordered by FDR and logFC, are shown. We also show some chosen biological markers ('MK'). There are NAs for the peak-associated columns if there was not a promoter peak in our set associated with that gene.
